# Supplementary material for: Content-rich biological network constructed by mining PubMed abstracts
Source: BMC Bioinformatics. 2004 Oct 8;5:147. doi: 10.1186/1471-2105-5-147 (PMC528731; doi:10.1186/1471-2105-5-147)
Supplement: Additional File 2 — The original results of the above study (non-essential files are deleted to keep the file size under the limit set by BMC bioinformatics). [file 1471-2105-5-147-S2.bz2 › chilibotAdditionalFile2/dip20/359ID9195914E5745/html/IL8_IL8RA.html]

 


 **IL8** and **IL8RA** 
  
Found 181 abstracts in PubMed, retrieved 20.  
 

 What does Google say? 
 PDF only 
| .edu only 

---

**Interactive relationship** (e.g. stimulation, inhibition, etc)

**Neutral relationship**- IL 8  [ **IL8** ]  Induced Migratory Responses through CXCR1  [ **IL8RA** ]  and CXCR2 Association with Phosphorylation and Cellular Redistribution of Focal Adhesion Kinase.  Ref: 12627953 Biochemistry, 2003
- IL 8  [ **IL8** ]  or CXCL8  [ **IL8** ]  activates the receptors CXCR1  [ **IL8RA** ]  IL 8RA and CXCR2 IL 8RB to induce chemotaxis in leukocytes, but only CXCR1  [ **IL8RA** ]  mediates cytotoxic and cross regulatory signals.  Ref: 12626541 J Immunol, 2003
- Interleukin 8  [ **IL8** ]  secreted by endothelial cells induces chemotaxis of melanoma cells through the chemokine receptor CXCR1  [ **IL8RA** ] .  Ref: 12738812 FASEB J< MedlineTA>FASEB J, 2003
- CXCR1  [ **IL8RA** ]  and CXCR2 mediate migratory activities in response to IL 8  [ **IL8** ]  and other ELR CXC chemokines e.g., GCP  [ **IL8** ]  2 and NAP  [ **IL8** ]  2.  Ref: 12627953 Biochemistry, 2003
- In addition, a significant reduction of IL 8  [ **IL8** ]  CXCL8  [ **IL8** ]  mediated cell migration was reported in the presence of anti CXCR1  [ **IL8RA** ]  monoclonal antibody.  Ref: 12614207 Br J Haematol, 2003
- When stimulated by tumor necrosis factor alpha, the nonresponsive WM35 melanoma cells synthesize a higher level of CXCR1  [ **IL8RA** ]  and become chemotactic toward interleukin IL 8  [ **IL8** ] .  Ref: 12738812 FASEB J< MedlineTA>FASEB J, 2003

**Non-interactive relationship** (e.g. studied together, co-existance, homology, etc.)

- Thus in COPD, in addition to CXCL8  [ **IL8** ]  and CXCR1  [ **IL8RA** ] , CXCL5 and CXCR2 appear to play important roles in the airway neutrophilia characteristic of severe exacerbations.  Ref: 12857718 Am J Respir Crit Care MedAm J Respir Crit Care Med, 2003
- However, the contribution of polymorphisms of the IL 8  [ **IL8** ]  gene and its receptor genes CXCR 1  [ **IL8RA** ]  and CXCR  [ **IL8RA** ]  2 to human TB susceptibility remains untested.  Ref: 12870115 J Infect Dis, 2003
- In this study, we addressed the question of whether expression of the interleukin 8  [ **IL8** ]  receptors IL 8RA CXCR1  [ **IL8RA** ]  and IL 8RB CXCR2 is upregulated in H. pylori infected human gastric biopsy samples.  Ref: 12761119 Infect Immun, 2003
- To investigate the effects of chemokine receptor cross regulation in HIV 1 infection, monocytes and MAGIC5 and rat basophilic leukemia RBL 2H3 cell lines co expressing the interleukin 8  [ **IL8** ]  IL 8  [ **IL8** ]  or CXCL8  [ **IL8** ]  receptor CXCR1  [ **IL8RA** ]  and either CCR5 ACCR5 or CXCR4 ACXCR4 were generated.  Ref: 12594210 J Biol Chem, 2003
- Our data suggest that IL 8  [ **IL8** ]  directly enhanced endothelial cell proliferation, survival, and MMP expression in CXCR1  [ **IL8RA** ]  and CXCR2 expressing endothelial cells and regulated angiogenesis.  Ref: 12626597 J Immunol, 2003
- In CXCR1  [ **IL8RA** ]  RBL cells this treatment resulted in FAK phosphorylation, in similar levels to those induced by activating concentrations of IL 8  [ **IL8** ] .  Ref: 12627953 Biochemistry, 2003
- Both IL 8  [ **IL8** ]  receptors, CXCR1  [ **IL8RA** ]  and CXCR2, are expressed extensively in the fetal intestine.  Ref: 12633567 Cytokine, 2002
- The interleukin 8  [ **IL8** ]  IL 8  [ **IL8** ]  CXCL8  [ **IL8** ]  receptor CXCR1  [ **IL8RA** ]  was expressed in 12 12 patients, the CXCR4 in 6 12 patients four CD3 ve and two CD3 ve and the CXCR3 in 3 12 patients one CD3 ve and two CD3 ve.  Ref: 12614207 Br J Haematol, 2003
- The immunohistochemical analysis using specific antibodies demonstrated that positive staining for IL 8  [ **IL8** ] , CXCR1  [ **IL8RA** ]  and CXCR2 in surgically resected human pancreatic cancer was 5 5 and 65%, respectively.  Ref: 12632066 Int J Oncol, 2003
- Antibodies against either IL 8  [ **IL8** ]  or CXCR1  [ **IL8RA** ]  inhibit melanoma transendothelial migration in a coculture assay by 30%.  Ref: 12738812 FASEB J< MedlineTA>FASEB J, 2003
- By comparison with the NSC and S COPD groups, neutrophilia and gene expression for epithelial derived neutrophil attractant 78 CXCL5, interleukin 8  [ **IL8** ]  CXCL8  [ **IL8** ] , CXCR1  [ **IL8RA** ]  and CXCR2 were each up regulated in the E COPD group p&lt.  Ref: 12857718 Am J Respir Crit Care MedAm J Respir Crit Care Med, 2003
- OBJECTIVE We sought to investigate the in vivo effect of bradykinin on the expression of the IL 8  [ **IL8** ]  receptors CXCR1  [ **IL8RA** ]  and CXCR2 in nasal cells.  Ref: 12532104 J Allergy Clin Immunol, 2003
- We examined the expression of IL 8  [ **IL8** ]  and its two receptors CXCR1  [ **IL8RA** ]  and CXCR2 in 40 surgically resected human pancreatic cancer tissues and in three different human pancreatic cancer cell lines PANC 1, MIAPaCa 2 and Capan 2.  Ref: 12632066 Int J Oncol, 2003
- The stimulation of CXCR1  [ **IL8RA** ]  and CXCR2 by IL 8  [ **IL8** ]  concentrations that result in migratory activation induced focal adhesion kinase FAK phosphorylation in a G alpha i dependent manner.  Ref: 12627953 Biochemistry, 2003
- IL 8  [ **IL8** ]  activation of CXCR1  [ **IL8RA** ] , but not the IL 8  [ **IL8** ]  receptor CXCR2, cross phosphorylated CCR5 and CXCR4 and cross desensitized their responsiveness to RANTES regulated on activation normal T cell expressed and secreted CCL5 and stromal derived factor SDF 1 or CXCL12, respectively.  Ref: 12594210 J Biol Chem, 2003
- In the present study, we examined the expression of chemokine receptors CXCR1  [ **IL8RA** ]  and CXCR2 receptors for interleukin 8  [ **IL8** ]  and CCR5 receptor for RANTES regulated on activation, normal T cell expressed and secreted, macrophage inflammatory protein MIP 1alpha, and MIP 1beta in human endometrium.  Ref: 12606476 Biol Reprod, 2003
- There are at least two different IL 8  [ **IL8** ]  receptor types CXCR1  [ **IL8RA** ]  and CXCR2.  Ref: 12561382 Sheng Wu Yi Xue Gong Cheng Xue Za Zhi, 2002
- It is well known that chemokines and chemokine receptors, such as interleukin 8  [ **IL8** ]  and its receptors CXCR1  [ **IL8RA** ]  and CXCR2, play important roles in the pathogenesis of psoriasis.  Ref: 12823305 Clin Exp Dermatol, 2003
- Expression of the chemokine receptors CCR2, specific for MCP 1, and CXCR1  [ **IL8RA** ]  and CXCR2, specific for IL 8  [ **IL8** ] , were not altered by HCMV infection.  Ref: 12714640 Invest Ophthalmol Vis Sci, 2003
- The mutants generated showed that a for CXCL8  [ **IL8** ]  binding, the N terminus of CXCR1  [ **IL8RA** ]  and the second extra cellular loop of CXCR2 are determinant, the N terminus of CXCR2 is not sufficient and the transmembrane domain seven is probably involved.  Ref: 12628493 Biochem Pharmacol, 2003
